# Male Mating Behaviour Is Shaped by Previous Experience of Both Conspecific and Heterospecific Females in the Seed Bug *Lygaeus simulans*

**DOI:** 10.1002/ece3.72883

**Published:** 2026-01-04

**Authors:** Vicki L. Balfour, Cédric Aumont, Mia K. Corliss, David M. Shuker

**Affiliations:** ^1^ School of Biology University of St Andrews UK; ^2^ L'institut Agro Rennes‐Angers Rennes France

**Keywords:** context‐dependence, *Lygaeus simulans*, mate choice, mating decisions, mating failure, reproductive interference

## Abstract

Mating decisions are often context‐dependent. For example, choosy individuals may benefit from relaxing mate preferences if conspecific mates are scarce. However, prior experience of heterospecifics can also alter mating decisions, and this can influence the strength of species discrimination and/or sexual selection. Here, we investigate the effect of previous mating opportunities on the subsequent mating decisions of male *Lygaeus simulans* seed bugs, a species known both to experience reproductive interference (reproductive interactions with heterospecifics that are costly) and also male mate choice for larger females. We used a nested, hierarchical design whereby focal males were: (1) paired with a conspecific female or remained unpaired on day 1; (2) paired with a conspecific female, a heterospecific female, or were unpaired on day 6; (3) paired with a conspecific female or a heterospecific female on day 8. The sister species *L. equestris* provided the heterospecific partners. We found that males were less likely to mate with heterospecific *L. equestris* females if they had previously encountered a heterospecific, but only if copulation had not occurred during that encounter. Additionally, the willingness of males to copulate with conspecifics increased when males had prior conspecific experience, and decreased with prior heterospecific experience, suggesting that male pre‐copulatory mating decisions are plastic and can be influenced by experience of both con‐ and heterospecifics.

## Graphical abstract

We investigate the effect of previous mating opportunities with con‐ and heterospecifics on the subsequent mating decisions of male *Lygaeus simulans* seed bugs. We found that males were less likely to copulate with both con‐ and heterospecific females if they had previously encountered a heterospecific female, and more likely to copulate with conspecific females if they had previously encountered a conspecific female. This suggests that male pre‐copulatory mating decisions are plastic and can be influenced by experience of both con‐ and heterospecifics.

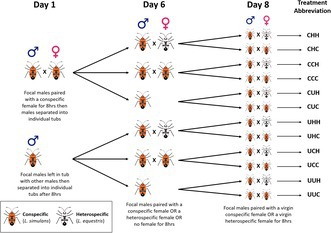

## 1 Introduction

Animal mating decisions are often context‐dependent (Cotton et al. 2006; Hebets and Vink 2007; Kim et al. 2009; Tinghitella et al. 2013; Ah‐King and Gowaty 2016). The costs and benefits of mating decisions are likely to vary depending on both external factors to the chooser (e.g., mate quality, mate density and predation risk: Jennions and Petrie 1997; Kvarnemo and Simmons 1999; Kim et al. 2009; Willis et al. 2011; Lindström and Lehtonen 2013) as well as intrinsic ones (e.g., reproductive status, age, and condition of the chooser: Kodric‐Brown and Nicoletto 2001; Hunt et al. 2005; Burley and Foster 2006; Cotton et al. 2006; Uetz and Norton 2006). For example, if mates are scarce, or only low‐quality mates are available, it should benefit choosy individuals to relax their mate preferences to reduce their chances of remaining unmated (Bleu et al. 2012; Scott et al. 2020). In addition, prior experience of potential mates can also influence future mate preferences. For instance, female *Schizocosa uetzi* wolf spiders that were exposed as sub‐adults to males of a particular phenotype went on to develop a preference for males with those familiar phenotypes (Hebets 2003).

Experience of potential mates may not only include experience of conspecifics, however (McDonald et al. 2019). Sexual interactions between heterospecifics are common in nature (Gröning and Hochkirch 2008). When a pair of individuals of different species engage in sexual activities, and these activities are costly to one or both species involved, this is known as reproductive interference (Gröning and Hochkirch 2008; Burdfield‐Steel and Shuker 2011; Shuker and Burdfield‐Steel 2017). Such activities can range from signal jamming (i.e., when signals from one species disrupt the sending or receiving of signals of another species) through to heterospecific matings and hybridisation (Gröning and Hochkirch 2008; Shuker and Burdfield‐Steel 2017). Reproductive interference is expected to be costly due to the time and energy wasted on such interactions and the potential loss of mating opportunities with conspecifics. Moreover, sexual interactions with heterospecifics can result in injury or even death (Fea et al. 2013; Shuker and Burdfield‐Steel 2017). Given that reproductive interference is often thought to be caused by incomplete species recognition (Gröning and Hochkirch 2008; but also see Shuker and Burdfield‐Steel 2017), we might expect mechanisms to be in place to improve species discrimination, for instance via learning from previous interactions with heterospecifics. One such example is from *Calopteryx* damselflies, whereby female *C. splendens* , which live in sympatry with *C. virgo* , learn to reject heterospecific males after experience of both con‐ and heterospecifics, whereas females in allopatric populations do not (Verzijden and Svensson 2016).

Here we investigated how previous mating opportunities, with both con‐ and heterospecific partners, affected the subsequent mating decisions of males of the seed bug *Lygaeus simulans*. *L. simulans* has a polygynandrous mating system, whereby both males and females mate multiply (Burdfield‐Steel and Shuker 2014). This species exhibits reproductive interference, as well as both pre‐ and post‐copulatory sexual selection (see below). In terms of reproductive interference, under laboratory conditions, heterospecific matings and hybridisation can occur between *L. simulans* and its sister species *L. equestris* (Burdfield‐Steel et al. 2015; Evans et al. 2015). Such pairings are asymmetrical, with male *L. simulans* recorded to copulate and hybridise with female *L. equestris* but not vice versa (Evans et al. 2015; though see Balfour et al. 2020a for exceptions to this with a mutant colour morph of *L. simulans* ). Reproductive interference has also been observed more widely in lygaeid bugs (Shuker et al. 2015). Why these heterospecific mating interactions arise is still unclear. Understanding how such interactions affect future mating decisions could not only give us insight into how speciation may have developed between these two species but also has implications for how reproductive interference could impact the strength of sexual selection.

With regard to within‐species pre‐copulatory sexual selection for *L. simulans* and *L. equestris* , the most consistent pattern across experiments is that larger females, and to some extent larger males, are more likely to mate, suggestive of both male and female mate choice (Dougherty and Shuker 2014; Greenway et al. 2017; Balfour et al. 2020b; Balfour et al. 2024a). As for post‐copulatory sexual selection, copulations involving larger females last longer and result in more offspring (Dougherty and Shuker 2014; Balfour et al. 2020b; Balfour et al. 2024a), and there is experimental evidence for sexual selection on the length of male genitalia in *L. simulans* (Dougherty et al. 2015). Moreover, in both *L. simulans* and *L. equestris* , many copulations (40%–60%) fail to result in offspring (Tadler 1999; Tadler et al. 1999; Micholitsch et al. 2000; Greenway and Shuker 2015; Greenway et al. 2017; Balfour et al. 2020b; Balfour et al. 2024a, 2024b; Balfour et al. 2024), a phenomenon termed cryptic mating failure (Greenway et al. 2015).

Understanding why mating failure occurs in these species is of particular interest. This is because we should expect this trait to be selected against, since a failed mating leads to no genes being passed onto the next generation (Greenway et al. 2015). Mating failure in *L. simulans* appears to be due to a lack of sperm transfer (Greenway et al. 2017), and in *L. equestris* , occurs more frequently in smaller females (Dougherty and Shuker 2014). This could be due to males preferring larger, more fecund females, and choosing, during copulation, to inseminate only these preferred females: a form of cryptic male choice (Bonduriansky 2001; Arnqvist 2014; Aumont and Shuker 2018). We do not yet know how reproductive interference might impact mating failure though, and so along with investigating the impact of previous mate experience and reproductive interference on mating decisions, we explored how such experiences might impact offspring production (i.e., the occurrence of mating failure).

**FIGURE 1:**
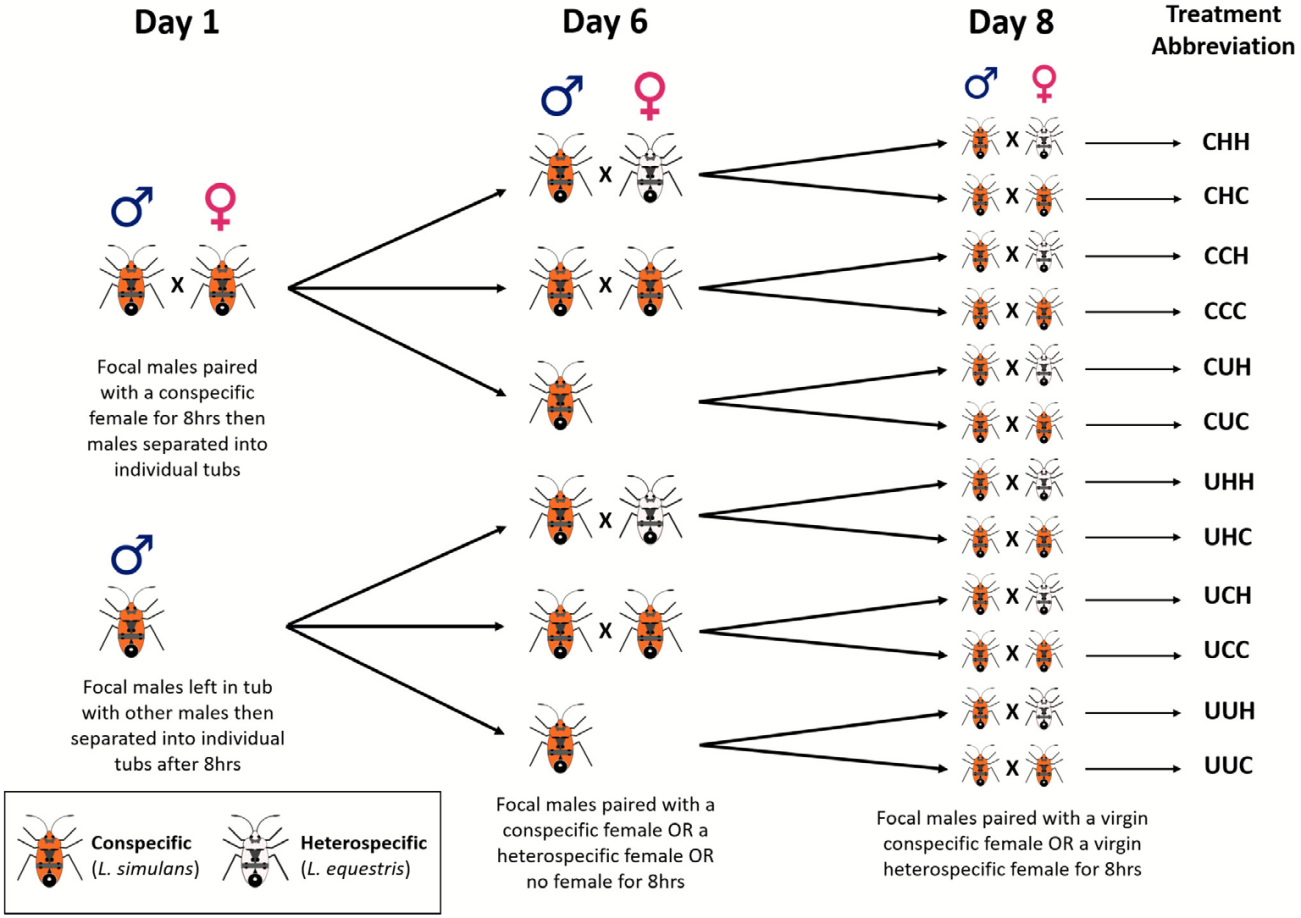
Graphical representation of the hierarchical design for the experiment. Focal males were either paired with a conspecific female on day 1 or were left in same‐sex tubs with other males then separated into individual tubs. On day 6 males were paired with either no female, a conspecific female or a heterospecific female. On day 8 males were paired with either a conspecific or heterospecific female. Treatment codes are shown for the 12 treatments (1st letter = day 1 treatment, 2nd = day 6, 3rd = day 8, C = paired with a conspecific, H = paired with a heterospecific, U = unpaired). Sample sizes per treatment combination: CHH = 43; CHC = 48; CCH = 48; CCC = 48; CUH = 47; CUC = 48; UHH: *N* = 43; UHC = 48; UCH = 44; UCC = 43; UUH = 46; UUC = 40.

Our experiment followed a nested, hierarchical treatment design, manipulating con‐ and heterospecific mating opportunities for focal male *L. simulans* across three separate days, using *L. equestris* as the heterospecific mating partner (Figure 1). We measured mating behaviour and mate preferences by quantifying willingness to mate (i.e., whether pairs copulated or not) and mating latency (pre‐copulatory sexual selection) as well as copulation duration and offspring production (post‐copulatory sexual selection).

We tested two hypotheses. First, we hypothesised that individuals may improve species discrimination following experience of interactions with heterospecifics. Therefore, we predicted that an individual that had encountered a heterospecific would be more likely to copulate with a conspecific partner, and less willing to copulate with a heterospecific partner than an individual with no previous heterospecific experience.

Second, we hypothesised that a lack of exposure over time to conspecifics is used as a cue of low mate availability. When mate availability is low, we expect male choosiness to decrease to reduce the risk of remaining unmated. Therefore, we predicted that males that had not had recent, or any, previous conspecific mating opportunities would be more likely to mate with a conspecific in the final trial (regardless of that female's quality, i.e., female body length). On the other hand, for males that had recently encountered conspecifics, and thus perceived mate availability to be high, we predicted that male choosiness would not decrease. As such, we predicted that males that had recently encountered conspecifics would be more likely to mate with a preferred female, i.e., a large female, and that post‐copulatory outcomes such as mating failure would be more strongly associated with this mate preference, i.e., mating failure would be less likely to occur with large females.

## 2 Materials and Methods

### 2.1 Study Species

*Lygaeus simulans* and *L. equestris* are sister species that have only been distinguished as separate species relatively recently (Deckert 1985). Both species occur in the Palearctic region. They share much of their range across Europe and Central Asia, being recorded in Spain through to Russia, although *L. simulans* remains absent from Scandinavia and North Africa (Deckert 1985; Solbreck et al. 1989; Gusev and Tararnikov 1992; Tadler et al. 1999; van der Heyden and Dioli 2019). The two sister species only differ externally in their antennal morphology and the male genital claspers (Deckert 1985). Otherwise, the species are behaviourally similar with the same polygynandrous mating system (Burdfield‐Steel and Shuker 2014). There is no apparent courtship in these species, and copulations are initiated by males climbing onto females and attempting to insert their intromittent organ. It is unclear to what extent males and/or females are in control of whether genital coupling occurs, with sexual conflict over mating being perhaps the best description (Arnqvist and Rowe 2005; Shuker et al. 2006). As both male and female body length have been associated with components of mating (see 1. Introduction), both sexes likely influence the outcome of mating interactions to some extent. Once pairs are coupled, they move into the characteristic back‐to‐back position for copulation. Copulations in these species can be long, lasting up to 24 h (Ludwig 1926; Kugelberg 1973), and these long couplings are thought to act as a form of male mate guarding (Sillén‐Tullberg 1981). A minimum copulation duration of 30 min is required for sperm transfer (Ludwig 1926; Gschwentner and Tadler 2000).

### 2.2 Husbandry

*Lygaeus simulans* were collected in Tuscany, Italy, in 2008 and 2009, and transferred to the Shuker Lab at the University of St Andrews. *L. equestris* were collected from Sicily in 1996 and have been maintained in a lab culture thereafter (Shuker et al. 2006). Therefore, the two populations used in this study are allopatric. The bugs were kept in population cages (30 × 15 × 15 cm plastic boxes) and provided with an *ad libitum* supply of dehusked organic sunflower seeds, cotton wool for habitat, and two cotton‐plugged tubes of distilled water (25 mL), which were changed once a week (all water provided to the bugs mentioned below was likewise distilled water). The bugs were kept in an incubator at 29°C on a 22:2 h light: dark cycle to prevent the onset of reproductive diapause. Humidity was neither measured nor controlled, but we have successfully reared and studied these bugs in the laboratory for more than two decades, and the provided water appears sufficient in terms of providing both a suitable water source and a suitably humid micro‐climate. A minimum of two replicate population cages were kept at any one time for each species. New population cages were created by transferring around 50 bugs from each instar from two separate population cages into a new cage approximately every 6–10 weeks, to maintain gene flow and limit inbreeding depression in the stocks.

We obtained experimental bugs by collecting late instar nymphs from population cages and transferring them, using an aspirator, to nymph boxes (20 × 10 × 8 cm plastic boxes) supplied with a cotton‐plugged water tube (25 mL), an *ad libitum* supply of sunflower seeds, and a piece of cotton wool for habitat. *L. simulans* and *L. equestris* individuals were housed separately.

To ensure that all bugs used in the following experiment were unmated, we checked the nymph boxes every 2–3 days for newly‐eclosed adults (adults become sexually mature at around 6–7 days post‐eclosion; Evans 2011). These were separated by species and sex into same‐sex tubs (108 × 82 × 55 mm plastic deli tubs), hereafter called collection tubs, with a maximum of 10 individuals per tub. We provided each tub with an *ad libitum* supply of sunflower seeds, a cotton‐plugged water tube (7 mL), and a piece of cotton wool for habitat.

### 2.3 Effect of Previous Mating Opportunities on Male Mating Decisions

To test the effect of previous mating opportunities with conspecifics and heterospecifics on subsequent mating decisions for male *L. simulans* , we provided male *L. simulans* with varied opportunities to mate with *L. simulans* and *L. equestris* females over eight days (Figure 1). The timescale chosen was for experimental convenience; however, this should have little impact on the behaviour of the bugs as the lifespan of the bugs under laboratory conditions is 2–3 months post‐eclosion (Shuker et al. 2006; Balfour et al. 2018). The experiment was nested in design (Figure 1). The treatments were hierarchical in their structure and essentially represented three nested treatment levels: focal males were (1) paired with a conspecific *L. simulans* female or no female on day 1; (2) paired with a female *L. simulans* , a female *L. equestris* or no female on day 6; (3) paired with a female *L. simulans* or a female *L. equestris* on day 8. This resulted in 12 treatment combinations, varying encounter rate and experience of heterospecifics (see Figure 1). We spread the treatments across the three months of the experiment, carrying out multiple treatments per experimental day. The treatment codes were designated as follows: the first letter denotes the treatment on day 1, the middle letter the treatment on day 6, and the last letter the day 8 treatment (C = paired with a conspecific, H = paired with a heterospecific, U = unpaired), giving us CHH, CHC, CCH, CCC, CUH, CUC, and UHH, UHC, UCH, UCC, UUH, UUC. Sample sizes for each treatment combination ranged from 40 to 48, with a total of 546 males used in 1193 intra‐ and interspecific crosses (see Figure 1 for the sample sizes for each treatment combination). Note that the data collected were not blinded relative to treatment, as the experimenter needed to know the treatments when setting up the mating trials each day, so that the bugs were paired up correctly. The effects of unblinded experimental observation can include unconscious bias when scoring behaviours or other variables, although we note here that we had 12 treatment combinations involving more than 1100 mate trials. With multiple mate trials performed simultaneously per day, keeping track of treatment combinations in terms of specific (unconsciously expected) trial outcomes would be difficult. Moreover, some of the data were collected after mating trials (including body size data and production of eggs and nymphs), where relevant containers were identified only by the unique replicate number, not the treatment combination, and so these data were collected blind to all details except replicate number.

#### 2.3.1 Experimental Protocol

Due to the number of bugs required for the experiment, females were re‐used. Some of these females were unmated (8–13 days post‐eclosion, collected from nymph boxes as described above), and others had previously been given the opportunity to copulate with focal males. After females were used in a mating trial, they were housed in “pre‐exposed” tubs that had the same specifications as collection tubs. For these females, female ID was not recorded. We reused these females up to 10 days after the first time they were presented to a focal male. Both unmated and “pre‐exposed” females were randomly allocated to males across the experiment for the day 1 and day 6 mating trials. We randomly allocated these females, blind to previous usage, to males across the experiment. There was a minimum of a two‐day gap between females being reused.

We acknowledge that mating decisions may be influenced by both focal and non‐focal individuals and the mating status of each (i.e., copulations are more likely to occur with mated than unmated females, either due to male preferences or mated females being more willing to copulate: Balfour et al. 2020b). However, in the wild, the bugs should happen upon both mated and unmated individuals. Therefore, reusing non‐focal bugs should help to mimic natural settings, and since non‐focal individuals were randomly allocated, this should control for any effects due to mating status across the treatment combinations. We also note here that there appears to be no female refractory period in these species as females will immediately copulate again with the same or another male after a copulation is terminated (Dougherty 2015; V.L.B. pers. obs.).

On day 1, half of the focal *L. simulans* males were given the opportunity to mate with a randomly chosen conspecific *L. simulans* female by placing one male with one female in a Petri dish (55 mm diameter), using soft forceps, and allowing them to interact unobserved for 8 h. This was done under ambient laboratory conditions. We did not observe or record copulations at this point because the aim was to expose focal individuals to mates, rather than assess their responses. We separated any pairs still copulating after 8 h by gently brushing their genitalia with a paintbrush, a procedure that does not influence future mating attempts. All focal males were then placed in individual tubs (108 × 82 × 55 mm) with 15–20 sunflower seeds and a water tube (7 mL), hereafter referred to as individual tubs. The other half of the focal *L. simulans* males (6–8 days post‐eclosion) were left in their collection tubs with conspecific males for the duration of the mating trials. At the end of the 8 h trial, these males were then also placed in individual tubs.

On day 6, we randomly paired the focal *L. simulans* males with either a conspecific female, a *L. equestris* female, or no female, in Petri dishes for 8 h. The females used were again either unmated or from “pre‐exposed” tubs (see above). Like the day 1 pairings, the purpose of the day 6 pairings was to provide focal individuals with varied mating opportunities to then assess whether differences in day 1 and 6 experience influenced decisions on day 8. However, unlike on day 1, the bugs were observed on day 6, so that we could record whether any interactions were occurring with heterospecific partners. We therefore checked pairs every 15 min and scored for whether they were copulating or not. We allowed any pairs that stopped copulating after two consecutive checks or less (< 30 min) to copulate again. We separated any pairs that stopped copulating after three consecutive checks or more (> 30 min) because 30 min is the minimum length of time required for sperm transfer (Ludwig 1926; Gschwentner and Tadler 2000), and we wanted to avoid multiple inseminations as much as possible. Note that for analytical purposes, only pairs that engaged in copulation for > 30 min were counted as having copulated. Any pairs that copulated for < 30 min were recorded as unmated (we note that the proportion of pairs that engaged in copulation, but for < 30 min, was small: only 6% for both day 6 and day 8 pairings). After 8 h, we separated any pairs still in copula and returned all males to their individual tubs and the incubator. Any females used were likewise returned to “pre‐exposed” tubs.

On day 8, we paired focal *L. simulans* males with either an unmated conspecific *L. simulans* female (8–10 days post‐eclosion) or an unmated heterospecific *L. equestris* female (8–10 days post‐eclosion), in Petri dishes for 8 h, following the same protocol as on day 6. Unlike on days 1 and 6, all focal males were paired with a female on day 8 (Figure 1). When pairs were separated, we placed all focal males in labelled Eppendorf tubes and froze them at −18°C for later measurements. Any females from pairs not observed to copulate were also frozen in Eppendorf tubes at −18°C. We placed females that were observed copulating with focal males in individual tubs and returned them to the incubator to lay eggs for 7 days. Females can begin ovipositing within 24 h of copulation, though they can also lay infertile eggs without the need for copulation to occur (V.L.B. pers. obvs). We allowed females 7 days to lay eggs as this provides sufficient time for females to lay multiple clutches and is the length of time used in other comparable behavioural experiments on this species (Burdfield‐Steel 2014; Balfour et al. 2018, 2020a, 2020b, 2024a, 2024b, 2024).

On day 15, we removed females from their individual tubs and euthanised them by freezing them in Eppendorf tubes at −18°C. We then scored tubs for the presence or absence of eggs. We discarded any tubs without eggs, whereas tubs with eggs were returned to the incubator for a further 7 days. As both mated and unmated females lay unfertilised eggs, it was necessary to keep eggs and allow them to hatch to assess whether mating failure had occurred and how many eggs had been fertilised. In *L. equestris* most fertilised eggs hatch 5–7 days after they are laid (Burdfield‐Steel 2014), and *L. simulans* appear to be very similar (V.L.B. pers. obs.). On day 22, we froze all remaining tubs at −18°C for a minimum of 24 h before counting any nymphs present.

#### 2.3.2 Measurements

Measurements of pre‐copulatory sexual selection taken on day 8 included whether pairs engaged in successful copulation (observed in copula for 3 consecutive checks or more), and the latency to successful copulation (time until pair was first observed in a copulation lasting 3 consecutive checks or more). Post‐copulatory measurements taken included copulation duration (if the pair was observed in copula for 3 or more consecutive checks during the mating trial, the copulation duration was calculated as the time of the last check observed in copula minus the time of the first check observed in copula), occurrence of mating failure (true if no nymphs were produced), and the number of nymphs produced.

Finally, we measured the body length of all the bugs after thawing using a dissecting microscope fitted with an eyepiece micrometre. We measured the length from the tip of the snout to the tip of the wings, dorsal side up. We re‐measured 68 bugs (34 females, 34 males), blind to the original measurements, to check measurement reliability. Our measurements were highly repeatable (intra‐class correlation coefficient: *r* = 0.974; one‐way ANOVA: F_67,68_ = 62.41, *p* < 0.001; Lessells and Boag 1987).

#### 2.3.3 Analysis

All statistical analysis was done using R statistical software version 4.4.3 (R Core Team 2025). We used a nested Generalised Linear Model (GLM) approach to assess the impact of current and previous mate experience on the different measured mating outcomes in the final mate trials (i.e., the response variables come from the final mate trials on day 8). We nested day 8 experience within day 6 experience, and day 6 experience within day 1 experience, denoted as: Day 1/Day 6/Day 8. Day 1 considers the effect of day 1 experience (paired with a conspecific female, or no female) on the final mating outcomes, Day 1/Day 6 considers the effect of day 6 experience (paired with a conspecific female, a heterospecific female, or no female) on the final outcomes whilst taking into account day 1 experience, and Day 1/Day 6/Day 8 considers the effect of day 8 experience (paired with a conspecific female, or a heterospecific female) on the final outcomes whilst taking into account both day 6 and day 1 experience. Significant results at these different levels of nesting inform us of the impact that these different experience combinations have on the measured mating outcomes observed in the final trials.

We performed three nested GLMs, with a binomial distribution and logit link function, to test the effect of current (day 8) and previous (day 1 and day 6) mate experience on (i) whether pairs copulated (as a binary yes/no outcome for each trial), (ii) whether pairs engaged in copulation within the first 15 min of the trial (i.e., mating latency) and (iii) offspring production/mating failure (again as a binary yes/no outcome for each trial).

We then made more specific comparisons to test the role of con−/heterospecific experience and its timing in more detail. To try and answer our first hypothesis (individuals improve species discrimination following experience of interactions with heterospecifics), we used binomial GLMs to test whether having a previous heterospecific encounter or not affected the likelihood of copulation occurring with (i) a heterospecific on day 8 (CHH & UHH vs. CCH, CUH, UCH & UUH), and (ii) a conspecific on day 8 (CHC & UHC vs. CCC, CUC, UCC & UUC). We also used binomial GLMs to test, for males paired with a heterospecific on day 6, whether engaging in successful copulation (3 or more consecutive checks) with that heterospecific on day 6 affected the chance of copulation occurring on day 8 when paired with either (i) a conspecific (treatments CHC & UHC) or (ii) a heterospecific female (treatments CHH & UHH).

To try and answer our second hypothesis (a lack of exposure over time to conspecifics is used as a cue of low mate availability), when a focal male was paired with a conspecific female on day 8, we used binomial GLMs to test (i) the likelihood of copulation occurring and (ii) whether mating failure occurred, depending on whether focal bugs had previous conspecific mating opportunities or not (CUC, CHC, CCC & UCC vs. UHC & UUC), and whether there was an interaction between this and female body length. We also used binomial GLMs to test the likelihood of copulation occurring when a focal male was paired with a conspecific female on day 8, when focal males had (ii) one or two previous conspecific mating opportunities (CHC, CUC & UCC vs. CCC), and (iii) a two or six day delay between conspecific encounters (CUC vs. UCC). Additionally, binomial GLMs were used to test the association between female body length and (i) copulating or not on day 8, and (ii) the likelihood of having offspring following copulation.

We then used nested linear models (LMs) with a Gaussian error distribution to test the effect of previous mating opportunities on (i) copulation duration in the final day 8 mating trials and (ii) the number of offspring subsequently produced. We also used a LM to test the likelihood of copulation occurring when a focal male was paired with a conspecific female on day 8, depending on whether focal bugs had previous conspecific mating opportunities or not (CUC, CHC, CCC & UCC vs. UHC & UUC), and whether there was an interaction between this and female body length. Additionally, an LM with a Gaussian distribution was also used to test the relationship between copulation duration and the number of offspring produced for (i) conspecific females and (ii) heterospecific females. Finally, LMs with a Gaussian distribution were used to test the relationship between female body length and (i) copulation duration and (ii) the number of offspring produced. In the results section, all means are presented ± their standard error. All terms in our GLMs and LMs were tested using a “type II” sums of squares approach, with an ‘F' test for Gaussian distributions or a likelihood ratio test (presented as *χ* ^2^) for binomial distributions (these tests use the *car* package, Fox and Weisberg 2019).

## 3 Results

**FIGURE 2:**
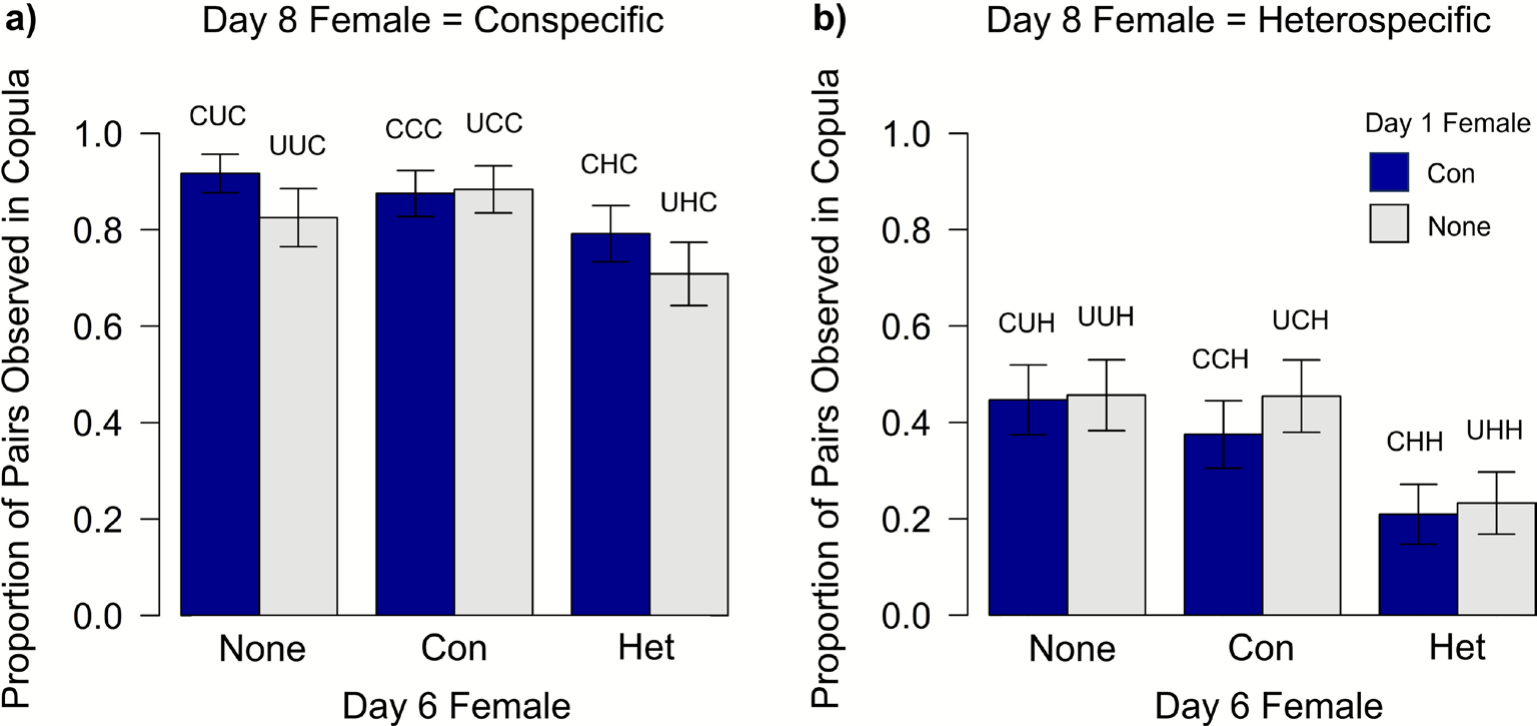
Proportion of (a) conspecific and (b) heterospecific pairs observed in copula for > 30min on day 8 depending on males' previous mate experience. The female the focal male was paired with on day 6 was either a conspecific (Con), a heterospecific (Het), or the focal male was not paired with a female on day 6 (None). Blue bars indicate males that were paired with a conspecific female on day 1, and grey bars represent males that were not paired with a female on day 1. Error bars represent the standard error. Treatment codes are given above each bar (1st letter = day 1 treatment, 2nd = day 6, 3rd = day 8, C = paired with a conspecific, H = paired with a heterospecific, U = unpaired).

Previous experience did influence subsequent male *L. simulans* pre‐copulatory mating decisions, an effect associated with encountering heterospecifics (Figure 2). In particular, males that had previously encountered a heterospecific female on day 6 were less willing to mate with both con‐ and heterospecific females on day 8.

### 3.1 Pre‐Copulatory Sexual Selection

For male *L. simulans* , whether or not a male copulated with a female in the final trial depended on both its experience on day 6 and the species identity of its partner on day 8 (Day 1: *χ* ^2^_1_ = 0.21, *p* = 0.65; Day 1/Day 6: *χ* ^2^_4_ = 12.49, *p* = 0.014; Day 1/Day 6/Day 8: *χ* ^2^_6_ = 140.2, *p* < 0.0001; Figure 2). A total of 328 out of 546 males copulated (60.1%) on day 8. Males were more likely to copulate with a conspecific than a heterospecific in the final trial (83.3% vs. 36.5%).

**FIGURE 3:**
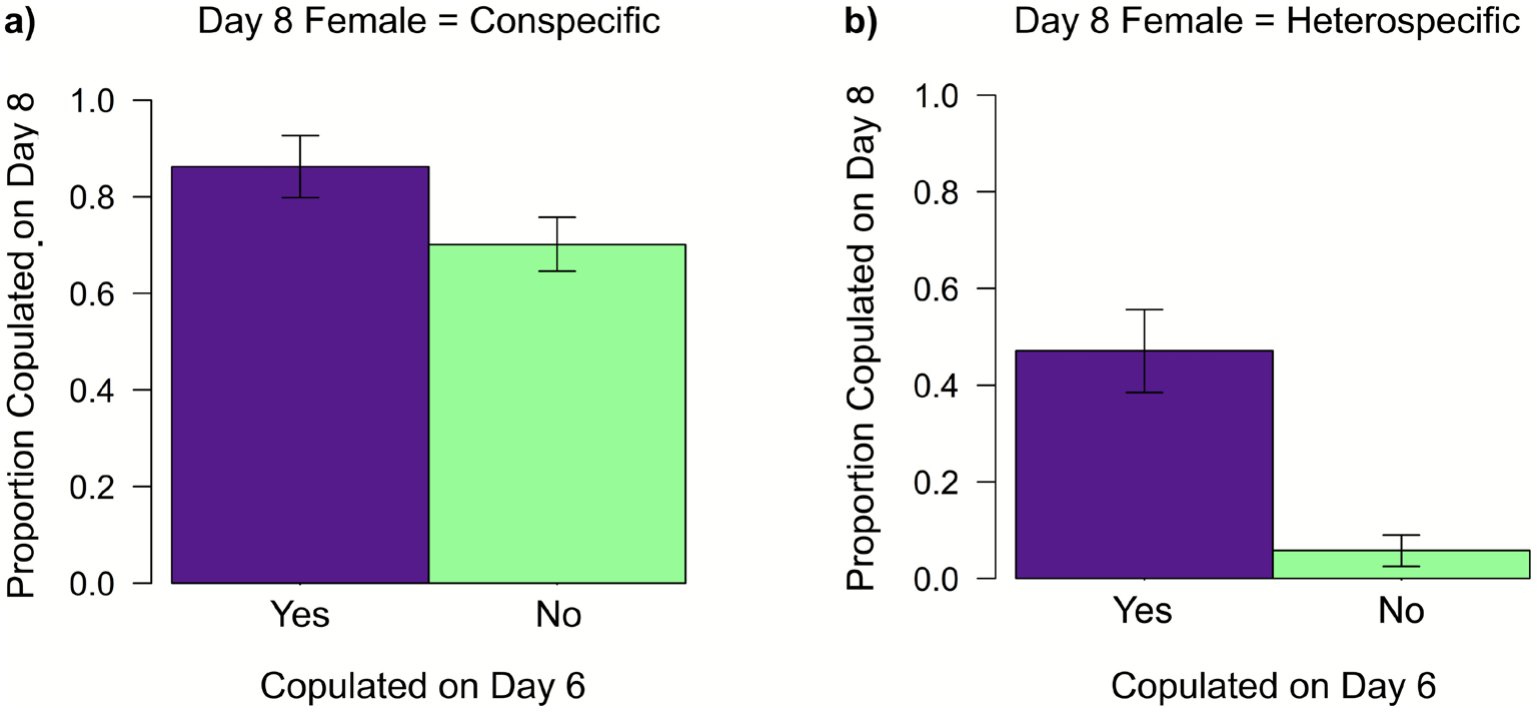
Proportion of males that copulated for > 30min on day 8, depending on whether that male had copulated with a heterospecific female (purple bar) or not (green bar) on day 6, for focal males paired with a heterospecific female on day 6 and (a) a conspecific female on day 8 (treatments = CHC, UHC), (b) a heterospecific female on day 8 (CHH, UHH). Standard errors are given on each bar.

Let us consider our first hypothesis i.e., that species discrimination improves with heterospecific encounters. One prediction was that males should be less willing to copulate with heterospecifics following an encounter with a heterospecific. We found that males were half as likely to copulate with a heterospecific female on day 8 if their previous encounter had also been with a heterospecific female compared to males naïve to heterospecifics (22.1% [CHH, UHH] vs. 43.2% [CCH, CUH, UCH, UUH]: *χ* ^2^_1_ = 11.87, *p* = 0.0006; Figure 2b). However, this relationship depended on whether a male had successfully copulated with the heterospecific female it encountered on day 6. Males were far less likely to copulate with a heterospecific on day 8 if they had not copulated with the heterospecific they encountered on day 6, compared to males that did copulate with the heterospecific they encountered on day 6 (5.8% vs. 47.1%: *χ* ^2^_1_ = 20.88, *p* < 0.0001; Figure 3b). On the other hand, males that did copulate with a heterospecific on day 6 were just as likely to copulate with a heterospecific female on day 8 as males that had not encountered a heterospecific on day 6 (47.1% [CHH, UHH, copulated on day 6 = yes] vs. 43.2% [CCH, CUH, UCH, CUH]: χ^2^_1_ = 0.17, *p* = 0.68). Therefore, the males appear to only alter their preferences and discriminate against heterospecific females if they had previously encountered, but did not copulate with, a heterospecific female.

Further, we predicted from our first hypothesis that males should be more willing to copulate with conspecifics following an encounter with a heterospecific. We found that, contrary to our first hypothesis, males that were paired with a conspecific female on day 8 were less likely to copulate on day 8 when the previous day 6 encounter had been with a heterospecific (75.0% [CHC, UHC]) compared to those that were not (87.7% [CCC, CUC, UCC, UUC]: *χ* ^2^_1_ = 6.96, *p* = 0.008; Figure 2a). Whether copulation took place with the heterospecific female encountered on day 6, however, did not seem to impact this. Males tended to be more likely to copulate with a conspecific on day 8 when they had copulated with a heterospecific female on day 6, compared to males that experienced a heterospecific female on day 6 but did not copulate, but this trend was not significant (86.2% vs. 70.1%; *χ* ^2^_1_ = 3.01, *p* = 0.083; Figure 3a).

Our second hypothesis was that a lack of exposure to conspecifics is used as a cue of low mate availability and thus should decrease male choosiness. Previous mating opportunities with a conspecific did slightly increase the likelihood of a male copulating with a conspecific on day 8 compared to males with no previous conspecific female encounters (86.6% [CCC, CHC, CUC, UCC] vs. 76.1% [UHC, UUC]: *χ* ^2^_1_ = 4.88, *p* = 0.027). However, there was no interaction between whether a male had previously encountered a conspecific or not and female length on the likelihood of copulation occurring (*χ* ^2^_1_ = 1.34, *p* = 0.247), which suggests that males were not more selective regarding any mate preferences when females were less scarce, contrary to our predictions for our second hypothesis. Moreover, the number of previous conspecific encounters did not matter (one previous encounter = 86.3% copulated on day 8: two previous encounters = 87.5%: *χ* ^2^_1_ = 0.04, *p* = 0.836) and having a longer delay between conspecific encounters (2 days for treatment combination UCC vs. 6 days for treatment combination CUC) also did not influence a male's propensity to copulate with a conspecific on day 8 (*χ* ^2^_1_ = 0.28, *p* = 0.6).

We next investigated whether previous experience impacted the mating latency of pairs that copulated on day 8. Whether a pair engaged in copulation in the first 15 min of the trial depended on the species of the female, with conspecific pairs quicker to initiate copulation (70.3% of pairs in first 15 min) than heterospecific pairs (60.6%; Day 1/Day 6/Day 8: *χ* ^2^_6_ = 13.84, *p* = 0.031). However, there were no effects of previous mating opportunities on mating latency (Day 1: *χ* ^2^_1_ < 0.001, *p* = 0.98; Day 1/Day 6: *χ* ^2^_4_ = 3.05, *p* = 0.55).

We also considered how female body length affected mating outcomes, since this is a trait associated with female quality (i.e., in insects, larger females are expected to be of higher quality than smaller females; e.g., see Honěk 1993). Considering only day 8 experience, and not previous experience, males were more likely to copulate with larger females on day 8, when the female species (i.e., conspecific or heterospecific) was included in the model (female length: *β* = 0.56 ± 0.32, *χ* ^2^_1_ = 5.73, *p* = 0.017; female species: *χ* ^2^_1_ = 75.4, *p* < 0.0001; interaction: *χ* ^2^_1_ = 0.003, *p* = 0.96). We note that the overall effect size for the association between female body length and mating success is small though. Indeed, when tested separately, neither the female *L. equestris* (*χ* ^2^_1_ = 2.63, *p* = 0.105) nor the female *L. simulans* (*χ* ^2^_1_ = 3.09, *p* = 0.079), body length influenced whether a male copulated with a heterospecific or conspecific (respectively) on day 8 or not. This suggests that our initial result that males are more likely to copulate with larger females is driven by males preferring to copulate with *L. simulans* females that are larger (mean body length = 11.47 ± 0.02 mm) than *L. equestris* females (mean body length = 11.00 ± 0.03 mm; F_1,527_ = 148.5, *p* < 0.0001).

### 3.2 Post‐Copulatory Sexual Selection

**FIGURE 4:**
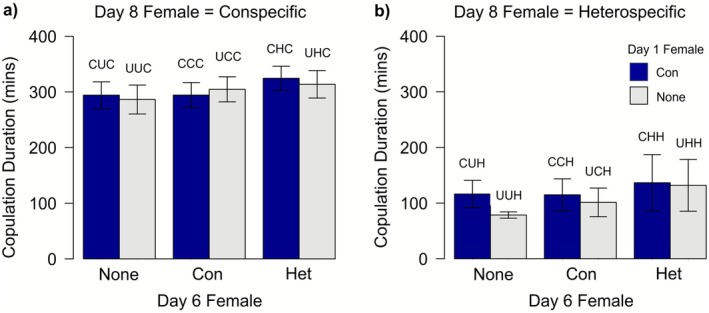
Mean copulation duration for (a) conspecific and (b) heterospecific pairs observed in copula for > 30min on day 8 depending on males' previous mate experience. The female the focal male was paired with on day 6 was either a conspecific (Con), a heterospecific (Het), or the focal male was not paired with a female on day 6 (None). Blue bars indicate males that were paired with a conspecific female on day 1, and grey bars represent males that were not paired with a female on day 1. Error bars represent the standard error. Treatment codes are given above each bar (1st letter = day 1 treatment, 2nd = day 6, 3rd = day 8, C = paired with a conspecific, H = paired with a heterospecific, U = unpaired).

The mean copulation duration for males that mated on day 8 was 244.1 ± 8.9 min. For these males, copulation duration depended on the species of female with which each male was paired (conspecific female: mean = 302.8 ± 9.6 min; heterospecific female: mean = 108.5 ± 11.0 min; Day 1/Day 6/Day 8: F_6,316_ = 22.09, *p* < 0.0001; compare Figure 4a with Figure 4b), but was also influenced by previous mate experience to some extent on day 6 but not day 1 (Day 1: F_1,316_ = 1.28, *p* = 0.26; Day 1/Day 6: F_4,316_ = 2.61, *p* = 0.036; Figure 4). However, we again found no support for our second hypothesis, since copulation duration with conspecific females on day 8 was unaffected by whether a male had previously encountered a conspecific female or not (F_1,259_ = 2.75, *p* = 0.098) and there was no interaction between this and female length (F_1,259_ = 1.30, *p* = 0.255). Copulation duration was, however, positively correlated with female body length for conspecific pairs (*β* = 73.2 ± 18.96, F_1,215_ = 14.93, *p* = 0.0001) but this was not the case for heterospecific pairings (F_1,97_ < 0.001, *p* = 0.99).

**FIGURE 5:**
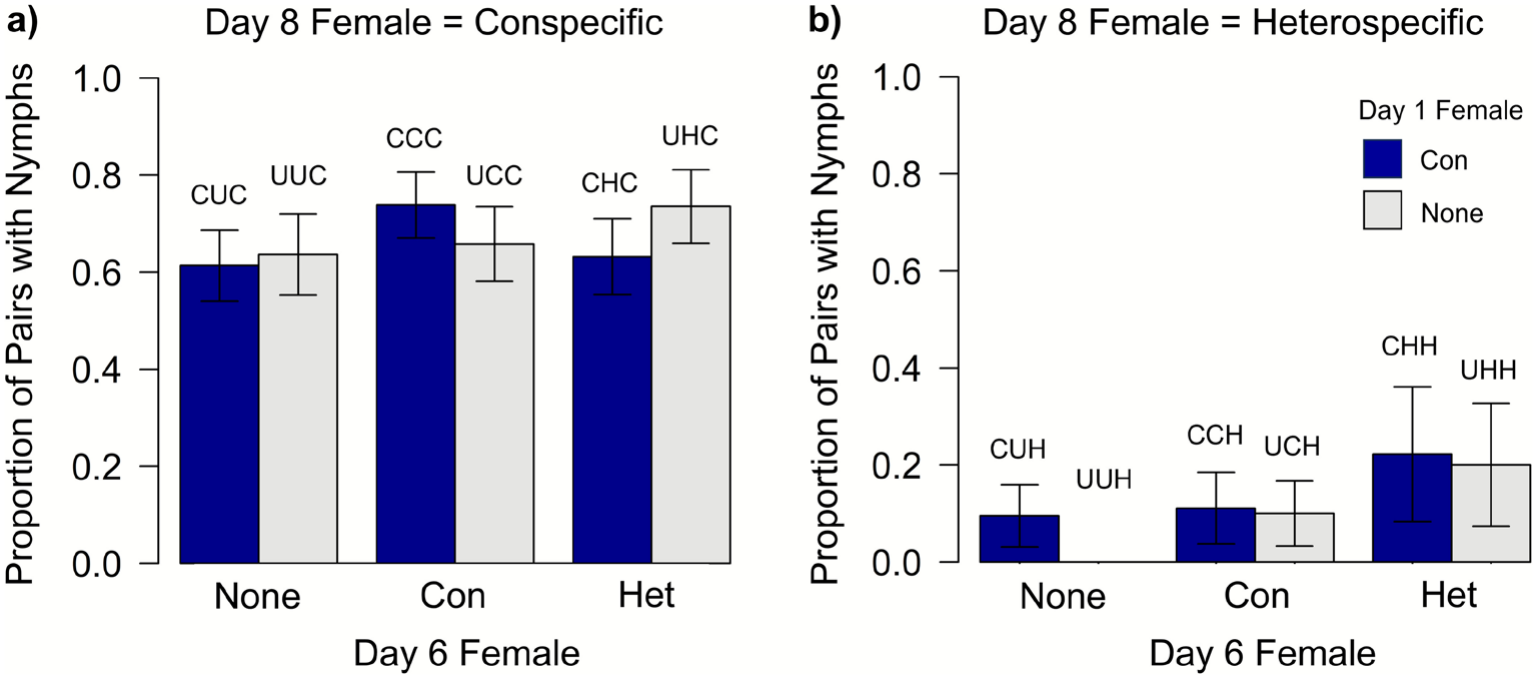
Proportion of pairs that had nymphs (i.e., did not experience mating failure) for (a) conspecific and (b) heterospecific pairs observed in copula for > 30min on day 8 depending on males' previous mate experience. The female the focal male was paired with on day 6 was either a conspecific (Con), a heterospecific (Het), or the focal male was not paired with a female on day 6 (None). Blue bars indicate males that were paired with a conspecific female on day 1, and grey bars represent males that were not paired with a female on day 1. Error bars represent the standard error. Treatment codes are given above each bar (1st letter = day 1 treatment, 2nd = day 6, 3rd = day 8, C = paired with a conspecific, H = paired with a heterospecific, U = unpaired).

Whether a copulation that occurred on day 8 resulted in mating failure (i.e., no nymphs produced) did not depend on previous experience on day 1 nor day 6 (Day 1: *χ* ^2^_1_ = 0.31, *p* = 0.58; Day 1/Day 6: *χ* ^2^_4_ = 6.82, *p* = 0.15), but was impacted by the species of the day 8 partner female. Conspecific females were far more likely to produce offspring (66.8%) than heterospecific females (10.1%; Day 1/Day 6/Day 8: *χ* ^2^_6_ = 100.8, *p* < 0.0001; Figure 5). Again, there was no support for our second hypothesis as there was no relationship between mating failure and whether a male had encountered a conspecific female before (*χ* ^2^_1_ = 0.15, *p* = 0.697), nor was there an interaction between this and female length (*χ* ^2^_1_ = 2.34, *p* = 0.123).

For the females that did produce offspring, the number of offspring that a male sired also depended on female species, with heterospecific females producing fewer nymphs (mean = 34.1 ± 9.7) than conspecifics (mean = 62.9 ± 2.8; Day 1/Day 6/Day 8: F_6,152_ = 2.32, *p* = 0.046), but there was no effect of previous mating opportunities on male siring success (Day 1: F_1,152_ = 0.09, *p* = 0.76; Day 1/Day 6: F_4,152_ = 0.22, *p* = 0.92). Males that copulated for longer on day 8 were more likely to sire offspring (*χ* ^2^_1_ = 201.68, *p* < 0.0001), and they also sired more offspring. This was true for males paired with both conspecific (F_1,151_ = 12.55, *p* = 0.0005) and heterospecific females (F_1,8_ = 6.34, *p* = 0.036).

Finally, larger *L. simulans* females were more likely to have nymphs (*χ* ^2^_1_ = 4.65, *p* = 0.031) and had significantly more nymphs than smaller females (F_1,147_ = 29.45, *p* < 0.0001) but the same was not true of *L. equestris* females (likelihood of having nymphs: *χ* ^2^_1_ = 0.35, *p* = 0.56; likelihood of having more nymphs: F_1,8_ = 0.07, *p* = 0.80), though the latter result may be due to the small sample size (only *N* = 10 *L. equestris* females produced hybrid offspring).

## 4 Discussion

We found that previous experience changes mating behaviour in male *Lygaeus Simulans*. As such, *L. simulans* joins the growing list of species where mating behaviour and its outcomes are context‐dependent (e.g., vertebrates: Pizzari et al. 2003; Tinghitella et al. 2013; Gauthey et al. 2016; Sommer‐Trembo et al. 2017; invertebrates: Hunt et al. 2005; Hebets and Vink 2007; Kim et al. 2009; Bailey et al. 2010; Billeter et al. 2012; Addesso et al. 2014). Performing such experiments as these can be time‐consuming though and—as here—involve large sample sizes and complicated experimental designs. This can bring limitations, and in our case the main limitation was our having to re‐use randomly chosen and allocated females for some mate trials with males. By doing so, despite the randomised presentation of females described in 2. Materials and Methods, we will undoubtedly have introduced some extra experimental noise and non‐independence. While we hope that this variation is for the large part orthogonal to the variation in the traits across treatment combinations we are interested in, readers should take this into account as we discuss and interpret our results.

Our first hypothesis was that interactions with heterospecifics improve species discrimination. If this is the case, we expected males to be less likely to copulate with a heterospecific and more likely to copulate with a conspecific following a previous heterospecific encounter. In the present study, males were less likely to copulate with heterospecific *L. equestris* females if they had previously encountered a heterospecific female, suggesting that species discrimination may be plastic in *L. simulans* males and/or *L. equestris* females. Therefore, the negative consequences of reproductive interference could be reduced by males changing their behaviour following a heterospecific encounter. However, this behavioural change in males was itself context‐dependent. If the previous encounter with a heterospecific included copulating with that heterospecific (i.e., a more complete form of reproductive interference), then males were no more or less likely to copulate with a heterospecific in the future compared to males that had never encountered a heterospecific. Therefore, it might be that not engaging genitalia and entering copula when encountering a heterospecific reduces male interest in future heterospecific partners. This falls somewhat in line with our first hypothesis; however, the nature of this interaction dictates how males subsequently behave towards heterospecifics. Indeed, successful heterospecific pairings may actually maintain the likelihood of future hybridisation events. Alternatively, one might interpret this as some males (i.e., the males that did not copulate with the heterospecific they encountered on day 6, and of which only 5.8% went on to copulate with a heterospecific female on day 8) already having an aversion to heterospecific females that did not change during their second heterospecific encounter. As for the males that did copulate with a heterospecific on day 6, only 47.1% of these copulated during their second heterospecific encounter, suggesting a reduction in willingness to copulate with heterospecifics compared to their previous behaviour (i.e., 100% of these males previously copulated with a heterospecific). So irrespective of which way we interpret the results, one group of these males has changed its behaviour towards heterospecifics (in terms of reduced willingness to mate) following a heterospecific encounter.

We also investigated the effect that previous heterospecific encounters had on future conspecific encounters. We found that males that had previously encountered a heterospecific were also less likely to copulate with a conspecific on day 8 than males with no heterospecific experience. This is the opposite of what we expected. A potential explanation for this is that males become choosier following a heterospecific encounter to reduce the chances of incorrectly selecting a heterospecific mate (for a review of potential costs of reproductive interference, see Shuker and Burdfield‐Steel 2017). In various species of *Drosophila*, evidence indicates that individuals can learn to recognise heterospecifics (Dukas 2004, 2008; see also 1. Introduction). For example, *D. melanogaster* males that had previous experience courting *D. simulans* females spent 40% less time courting heterospecific females than conspecifics, whereas naïve males did not discriminate between con‐ and heterospecifics (Dukas 2004). Over time, such heterospecific aversions have the potential to lead to reproductive character displacement (e.g., *Calopteryx* damselflies Tynkkynen et al. 2004; Verzijden and Svensson 2016), population divergence, and ultimately reproductive isolation.

In *L. simulans* , the reduction in willingness to copulate with both con‐ and heterospecifics after encountering heterospecifics could be because the benefits of avoiding copulation with a heterospecific outweigh the costs of being more selective when choosing a conspecific partner. Given the overlap in ranges of the two species, being able to discriminate against and avoid mating with heterospecifics is likely to be beneficial, as heterospecific matings (or at least those recorded between male *L. simulans* and female *L. equestris* ) often fail to produce offspring or result in a lower number of offspring, and the hybrids have lower fitness than wild‐type individuals (Evans et al. 2015; Balfour et al. 2020a). In this study, only 10.1% of heterospecific copulations resulted in hybrid offspring, compared to 66.8% of conspecific matings resulting in offspring. We additionally note here that while the populations used in our experiment were allopatric, we may expect species discrimination to be even greater in sympatric populations, where the risk of reproductive interference between *L. simulans* and *L. equestris* would be present.

Our second hypothesis was that a lack of exposure over time to conspecifics is used as a cue of low mate availability and that low mate availability would lead to a decrease in male choosiness. When conspecifics are scarce, it is expected that individuals should lower their mate acceptance threshold to reduce the risk of remaining unmated (e.g., Kokko and Mappes 2005; Bleu et al. 2012; Scott et al. 2020). This means that not only might individuals be more willing to mate with conspecifics when encountered, but heterospecifics might also be considered as potential mates. For example, in the swordtail fish, *Xiphophorus birchmanni* , females spent more time associating with a heterospecific male when their last encounter with a conspecific male was 24 h ago, compared to when that encounter was immediately before the trial (Willis et al. 2011). The authors suggest that this implies a decrease in female choosiness when encounter rate is low (Willis et al. 2011). In terms of our experiment, we predicted that (i) males with less recent, fewer, or no previous conspecific mating opportunities would be more willing to accept a conspecific mate, and that (ii) choosiness in relation to female quality (in this case female length) would be stronger in males that had previously had conspecific mating opportunities. With the exception of males with no previous conspecific experience (see below), we found none of these to be true. Therefore, our results suggest that mate limitation (or encounter rate over this timeframe) might not be important for shaping mating decisions in *L. simulans* . We note however that generally we found high rates of mating with conspecific females, creating a possible “ceiling effect”. One possible reason for this is that the focal males had not experienced enough reproductive interactions to find repeated interactions costly enough to lead to greater choosiness.

That said, we did find that males that had previously encountered a conspecific tended to be more likely to engage in copulation with a conspecific female on day 8 than those that had not. This could potentially be a learned response whereby males, through learning or some other mechanism, either gain a preference for conspecific females or have their existing preference in some way reinforced. We note here, however, that this only led to a small increase in the proportion of pairs that engaged in copulation with a conspecific in the final trial (0.87) compared to males that had no previous conspecific experience (0.76; an increase of 14.5%). In comparison, males with previous heterospecific experience were less likely to copulate with a heterospecific on day 8 compared to males without previous heterospecific experience (proportion reduction of 0.43 to 0.22; a decrease of 48.8%). Therefore, we can say that the aversion response to heterospecifics following a heterospecific encounter (though only when successful copulation did not occur during this encounter) is much greater than the increased preference for conspecifics following a conspecific encounter, again accepting a likely ceiling effect on how much higher that latter can go. Even if the differences in magnitude are thus inflated to some extent, it still suggests that the benefits of learning to avoid heterospecifics are greater than benefits relating to increased preferences, or recognition, of conspecifics.

Our experiment also enabled us to further explore the occurrence of mating failure in these species. The failure to produce offspring despite having copulated, primarily due to insemination failure, is high in *L. simulans* and *L. equestris* (40%–60%: Tadler 1999; Tadler et al. 1999; Micholitsch et al. 2000; Greenway and Shuker 2015; Greenway et al. 2017; Balfour et al. 2020b; Balfour et al. 2024a, 2024b; Balfour et al. 2024). In our experiment, overall mating failure increased due to reproductive interference, but only in the sense that heterospecific couplings were less likely to result in offspring than conspecific couplings. In terms of mating experience, male mating history had no effect on mating failure arising from the day 8 trials, suggesting that reproductive interference has no impact on the chance of mating failure occurring with a future conspecific partner. In our experiment, mating failure was associated with copulation duration, with shorter copulations more likely to yield no offspring, in line with previous findings (Balfour et al. 2020b; Balfour et al. 2024a, 2024b; Balfour et al. 2024). Since copulations with heterospecific females were shorter than those with conspecific females, copulation duration (which itself could be driven by cryptic male choice) likely contributes to the higher mating failure rate in heterospecific pairings.

Finally, we additionally found evidence for cryptic male choice, a form of mate choice which occurs during or after copulation, whereby males bias resources, such as sperm or nuptial gifts, towards females of a favoured phenotype (Bonduriansky 2001; Arnqvist 2014; Aumont and Shuker 2018). Focal males copulated for longer with larger conspecific females, and these longer copulations resulted in females having (i) a higher probability of producing offspring, and (ii) a greater number of offspring, compared to pairings with shorter copulation durations. While this adds to the increasing evidence suggesting the occurrence of cryptic male choice in *L. simulans* (Balfour et al. 2024a, 2024b; Balfour et al. 2024), it does not conclusively rule out the possibility that these patterns are either partly or wholly driven by cryptic female choice instead (see also Balfour et al. *in review*).

In conclusion, we found that male *L. simulans* reduced their likelihood of mating with heterospecific *L. equestris* females if they had previous experience of a heterospecific, unless they had copulated with that heterospecific female. This suggests that species discrimination by males is plastic to some extent in this species and is mediated by whether individuals have previously engaged in interspecific matings. In addition, males were also more likely to mate with a conspecific after previously encountering a conspecific female, and less likely to mate with a conspecific after previously encountering a heterospecific female, showing that within‐species willingness to mate is also phenotypically plastic in males.

## Author Contributions

**Vicki L. Balfour:** data curation (equal), formal analysis (lead), methodology (supporting), writing – original draft (lead), writing – review and editing (lead). **Cédric Aumont:** data curation (equal), writing – review and editing (supporting). **Mia K. Corliss:** data curation (supporting), writing – review and editing (supporting). **David M. Shuker:** conceptualization (lead), formal analysis (supporting), methodology (lead), supervision (lead), writing – original draft (supporting), writing – review and editing (equal).

## Funding

This work was supported by the University of St Andrews.

## Ethics Statement

All work carried out complied with local and national animal welfare regulations. Our work involved the use of insects (*Lygaeus simulans* and *L. equestris* ), for which no review is necessary for carrying out experiments on. There are no welfare or environmental implications of the experimental design or procedures.

## Conflicts of Interest

The authors declare no conflicts of interest.

## Data Availability

The research data supporting this publication can be accessed at https://doi.org/10.17630/d9a60b78‐4def‐4637‐b89e‐f59df7edc855.
